# Relating atomic energy, radius and electronegativity through compression

**DOI:** 10.1039/d0sc06675c

**Published:** 2021-01-28

**Authors:** Martin Rahm, Paul Erhart, Roberto Cammi

**Affiliations:** Department of Chemistry and Chemical Engineering, Chalmers University of Technology SE-412 96 Gothenburg Sweden martin.rahm@chalmers.se; Department of Physics, Chalmers University of Technology SE-412 96 Gothenburg Sweden; Department of Chemical Science, Life Science and Environmental Sustainability, University of Parma Parma Italy

## Abstract

Trends in atomic properties are well-established tools for guiding the analysis and discovery of materials. Here, we show how compression can reveal a long sought-after connection between two central chemical concepts – van-der-Waals (vdW) radii and electronegativity – and how these relate to the driving forces behind chemical and physical transformations.

## Abstract 2

Compression is used to derive a long sought-after connection between two central chemical concepts – van-der-Waals (vdW) radii and electronegativity – and how these relate to the driving forces behind chemical and physical transformations.

## Graphical abstract

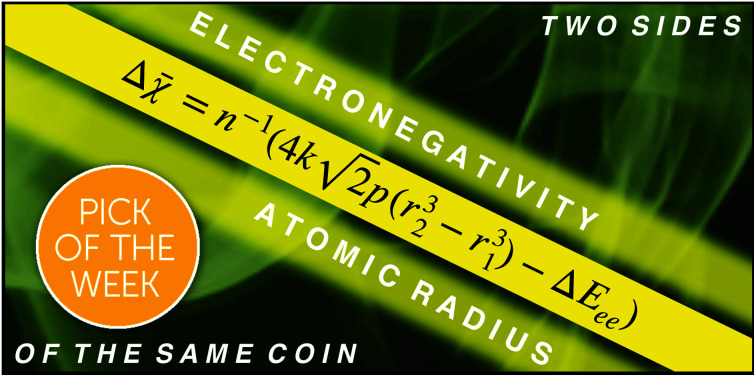

## Introduction

Atomic radii and electronegativity are often quintessential for how chemistry is rationalized.^1,2^ The history of quantifying the sizes of atoms under ambient conditions includes a large body of work, extending over the last one and a half-centuries (for a non-exhaustive summary of this history see ref. 3 and 4). One early motivation for attaining atomic and ionic sizes was to help understand X-ray diffraction patterns in terms of crystal structures,^5,6^ another to provide a rationalization for metallization.^7–9^ Today, a variety of definitions of atomic radii with well-known uses exists, including, *e.g.*, ionic,^10–12^ covalent,^6,13–17^ and vdW radii.^3,18–21^ Electronegativity is a similarly well-studied concept that can be defined in many ways (see, *e.g.*, ref. 22–29 and references therein).

A relationship is intuitively expected between electronegativity and radius: the size of an atom is determined by the distribution of electrons around its nucleus. The closer the electrons are to the nucleus, the more tightly they are bound, thus increasing the electronegativity of the atom. Pitzer pointed out the periodic behavior in the two atomic properties long ago^30^ and many others have relied on different definitions of atomic radii (usually covalent radii) and electrostatic relationships to define scales of electronegativity.^24,31–44^

Thus far, relationships between atomic radii and electronegativity have mostly been sought under ambient conditions and have been rationalized by comparing different atoms. In this work we take a different perspective and instead consider how electronegativity changes as the sizes of individual atoms are modified. The means by which we change the size of individual atoms is through physical compression.

There exist different frameworks through which electronegativity might be related to radii under compression. Garza *et al.*^45^ and Chattaraj and co-workers^46,47^ have, for example, relied on conceptual density functional theory to evaluate the electronegativity for a selection of atoms compressed by impenetrable spherical cavities. In a related work, Sen *et al.* have calculated the critical diameter at which spherical confinement causes ionization of some atoms.^48^ In this work, we rely on two revised scales of atomic vdW radii^3^ and electronegativity^29^ which have been extended to high pressure conditions (0–300 GPa).^4,49^

The scale of electronegativity used here is inspired by the work of Allen^26^ and is defined as the average electron binding energy as *T* → 0 K.^29,50^ This definition of electronegativity establishes a connection with the total energy of a system through an energy decomposition analysis:^51^

1 Δ*E* = −*n*Δ*

<svg xmlns="http://www.w3.org/2000/svg" version="1.0" width="12.769231pt" height="16.000000pt" viewBox="0 0 12.769231 16.000000" preserveAspectRatio="xMidYMid meet"><metadata>
Created by potrace 1.16, written by Peter Selinger 2001-2019
</metadata><g transform="translate(1.000000,15.000000) scale(0.013462,-0.013462)" fill="currentColor" stroke="none"><path d="M160 1000 l0 -40 240 0 240 0 0 40 0 40 -240 0 -240 0 0 -40z M240 840 l0 -40 -40 0 -40 0 0 -40 0 -40 -40 0 -40 0 0 -40 0 -40 40 0 40 0 0 40 0 40 80 0 80 0 0 -160 0 -160 -40 0 -40 0 0 -80 0 -80 -40 0 -40 0 0 -80 0 -80 -40 0 -40 0 0 -40 0 -40 80 0 80 0 0 80 0 80 40 0 40 0 0 80 0 80 40 0 40 0 0 -80 0 -80 40 0 40 0 0 -40 0 -40 40 0 40 0 0 -40 0 -40 40 0 40 0 0 40 0 40 40 0 40 0 0 40 0 40 -40 0 -40 0 0 -40 0 -40 -40 0 -40 0 0 40 0 40 -40 0 -40 0 0 80 0 80 -40 0 -40 0 0 40 0 40 40 0 40 0 0 120 0 120 40 0 40 0 0 40 0 40 40 0 40 0 0 80 0 80 -40 0 -40 0 0 -40 0 -40 -40 0 -40 0 0 -40 0 -40 -40 0 -40 0 0 40 0 40 -40 0 -40 0 0 40 0 40 -40 0 -40 0 0 -40z"/></g></svg>

* − Δ*E*_ee_ + Δ*E*_NN_,

where Δ*E* is the change in total energy over a chemical or physical transformation, *n* is the total number of electrons, Δ*χ̄* is the change in electronegativity (here defined as the average electron binding energy), while Δ*E*_ee_ and Δ*E*_NN_ are changes in the electrostatic repulsion between electrons (under the influence of exchange and correlation effects) and nuclei, respectively.

Eqn (1) can be recast to also partition relative enthalpies or free energies. Specifically, for compression at *T* → 0 K, we can write:

2 Δ*H* = −*n*Δ*χ̄* − Δ*E*_ee_ + Δ*E*_NN_ + Δ(*pV*),

where Δ(*pV*) describes changes in volume *V* and pressure *p*. The relationship between enthalpy, electronegativity, nuclear geometry, electron interactions, pressure, and volume provided by eqn (2) is exact within the Born–Oppenheimer approximation. We refer to eqn (2) as an “Experimental Quantum Chemistry” partitioning^50^ because it is, in principle, possible to estimate all of its terms directly or indirectly from a combination of thermal measurements, photoelectron spectroscopy, X-ray diffraction structure determination, and, for the Δ(*pV*)-term, equations of state. How does eqn (2) thus relate to atomic radii?

### The challenge of estimating atomic radii at high pressure

Pressure is a macroscopic observable, defined in terms of an ensemble of atoms. We can, therefore, in principle, relate the volume *V* of eqn (2) to an average atomic volume. Atomic volumes can, and have been, measured as a function of pressure using a variety of experimental techniques (see, *e.g.*, ref. 52–54). We note that Young has provided a schematic overview of atomic volumes at selected higher pressures obtained by an amalgamation of experiment and calculation on condensed phases.^53,54^ In spite of a wealth of equation-of-state data, a challenge arises when one tries to extract atomic radii from volumes of real materials. The challenge relates to structure and bonding and may be exposed most clearly by example: if we consider the atomic volume of hydrogen (at a given pressure) and attempt to translate such an experimental number to a radius, we obtain a value that is some average of a covalent and a vdW radii. The H atoms are bonded together in H_2_ molecules, which in turn have longer (non-bonded) distances between them. Similarly, if we look at atomic volumes of heavier elements, the radii extracted will correspond to metallically bonded radii, not vdW radii, and will moreover depend on (crystalline or liquid) structure. Approximations of vdW radii under pressure have been obtained in the context of the activation volume of some organic reactions, but not been systematically reported (see, *e.g.*, ref. 55). The only explicit and systematic experimental estimates of atomic radii at higher pressures that we are aware of are by Royce, who used the Wigner–Seitz definition to evaluate a selection of (metallically) bonded elements.^56^

A straightforward method for obtaining non-bonded radii from experimental atomic volumes is practically limited to noble gas elements: assuming that such atoms pack perfectly as hard spheres with a fraction of the total volume equaling 
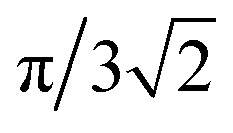
, pressure-resolved radii can be calculated as,3
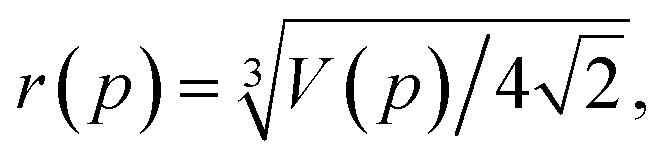
where *V*(*p*) is the atomic volume. We have calculated such radii for reference purposes.^4^ But what about vdW radii of atoms that readily engage in chemical bonding?

The challenge of non-bonded/vdW radii can be solved computationally by considering single atoms compressed by a homogeneous non-reacting environment.^4,49^ The properties – radii, electronegativity as well as ground state electron configurations – of such compressed atoms have been determined in the pressure range from 0 to 300 GPa through full potential relativistic density functional theory calculations combined with the eXtreme Pressure Polarizable Continuum Model (XP-PCM).^4,49,57,58^ We stress that by using this method we purposefully exclude the effects of both crystal structure and chemical bonding. The sizes of the atoms are in our model purely a consequence of isotropic non-reactive compression. Such computed non-bonded high-pressure radii are in excellent agreement with experimental compression isotherms for noble gas elements, when experimental radii are defined using eqn (3).^4,49^ Calculated non-bonded radii also correlate reasonably well with Wigner–Seitz radii of bonded metallic elements compiled from shock-wave experiments,^56^ but are, as expected, larger than such bonded radii.

## Results and discussion

**Fig. 1:**
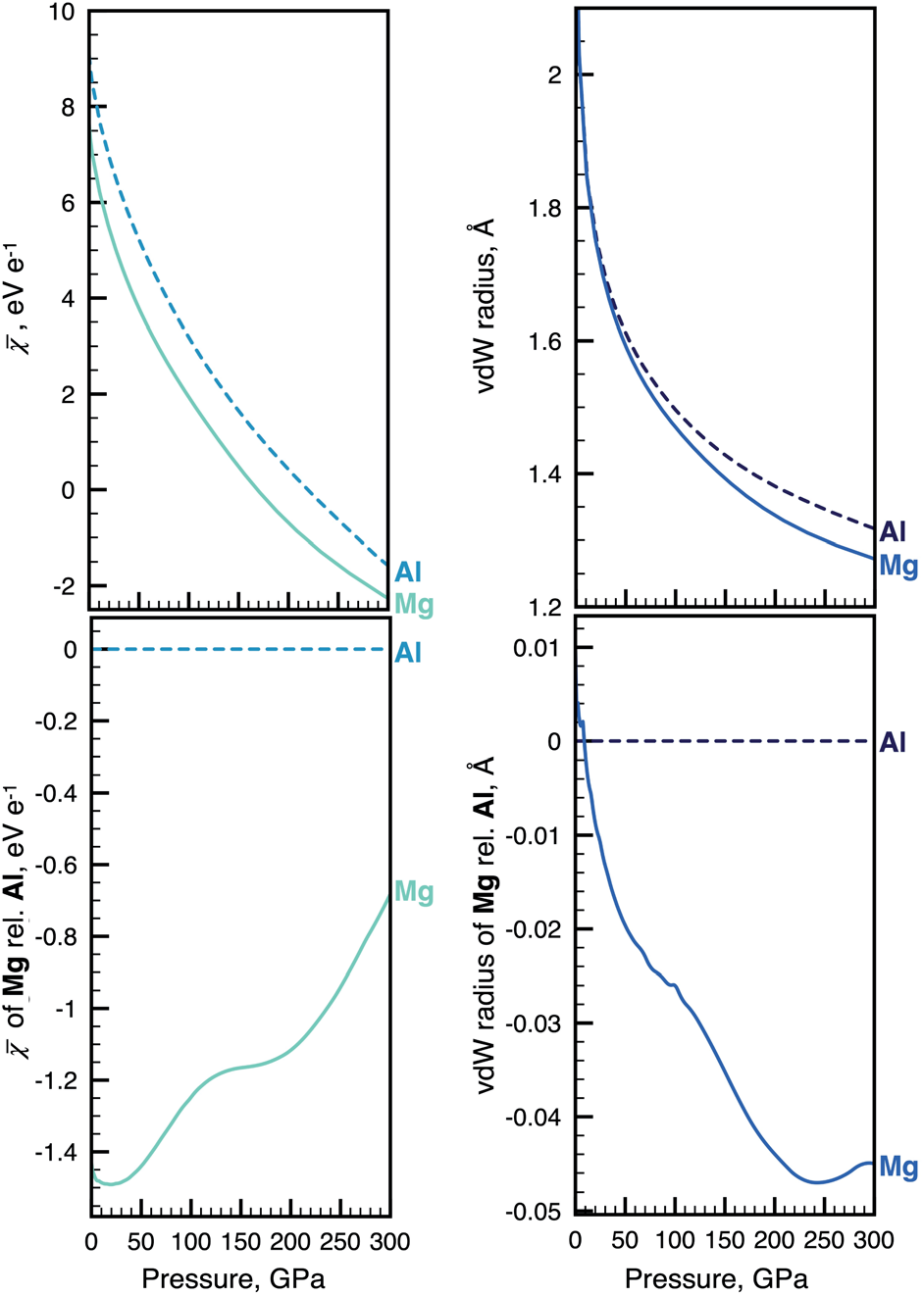
Example of the sometimes complex relationship between atomic radius and electronegativity. Absolute changes in *χ̄* (top left) and radius (top right) of Mg and Al during non-reactive compression. The same data for Mg is shown relative to Al for *χ̄* and radius at bottom left and right, respectively. The pressure evolution of radii and electronegativity for other atoms can be visualized with the AUP database.^59^

We will in what follows look at the evolution of electronegativity and radius computed for a selected combination of atoms. Our complete dataset of 93 atoms has been compiled into an interactive web application, the Atoms-Under-Pressure (AUP) database.^59^Fig. 1 shows an excerpt of our data and compares the change of the non-bonded radius and electronegativity of Mg and Al with pressure.

The top part of Fig. 1 implies a strong dependence of electronegativity on atomic radius – when individual atoms are compressed their size diminishes along with their electronegativity. This dependence is now quantified for any atom within our definition of these properties.^4,49^ We note that, because we consider compression of non-interacting atoms, conclusions drawn from our data can in instances appear at odds with related work where electronegativity is instead defined through the use of covalent radii or heats of reaction, such as in the work of Batsanov.^41,60^ The discrepancies occur in part because covalent radii may both increase and decrease in certain pressure ranges.^61^ In contrast, atomic volumes of non-bonded atoms (as well as bonded elements) monotonically decrease with compression. The bottom part of Fig. 1 shows how trends in the changes of radii and electronegativity can be opposite in a relative comparison between atoms. There remain 4277 binary combinations of atoms to consider and the interested reader is encouraged to perform other comparisons using the AUP database.^59^

### Relating atomic radius, electronegativity and energy

**Fig. 2:**
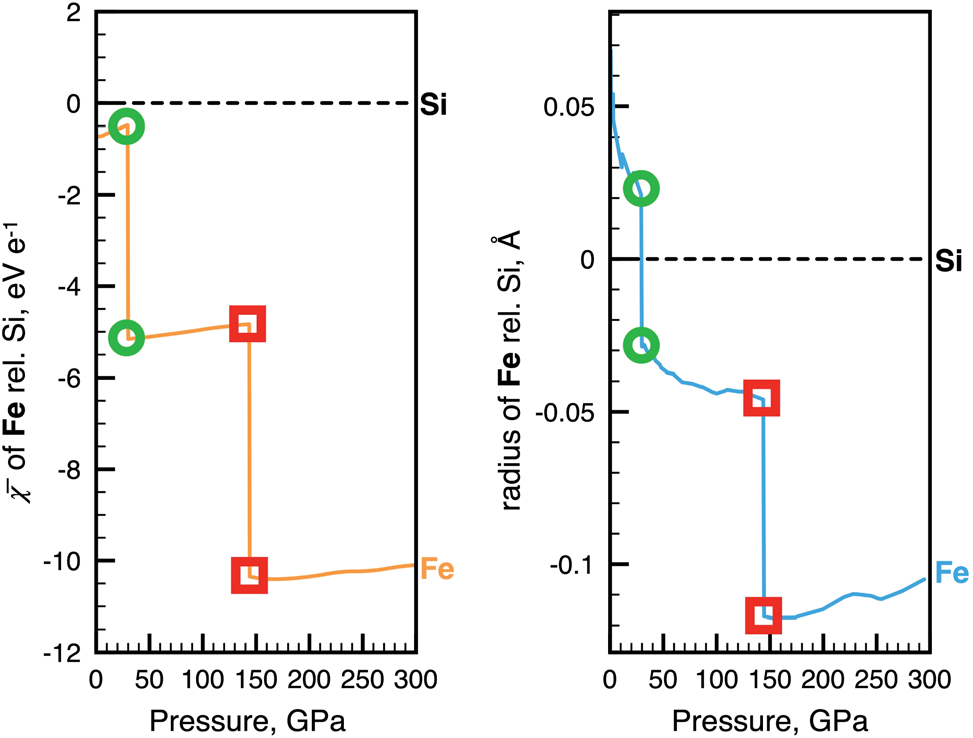
Examples of the relationship between atomic radius and electronegativity. Changes in *χ̄* (left) and radius (right) of Fe during non-reactive compression. The data is shown relative to Si. Green circles and red squares indicate two pressures, both at which the Fe atom can have either of two radii, two electronegativities and two energies. The two vertical drops coincide with [Ar]4s^2^3d^6^ (*S* = 2) → [Ar]4s^1^3d^7^ (*S* = 2) and [Ar]4s^1^3d^7^ → [Ar]3d^8^ (*S* = 1) ground state configurational transitions in the Fe atom. Slight jaggedness in the data for the radii is a computational artefact arising from extrapolation from a finite number of compression calculations. The pressure evolution of radii and electronegativity for other atoms can be visualized with the AUP database.^59^

The key we will use to establish a more rigorous relationship between radius and electronegativity is the phenomenon of pressure-induced changes to the ground state configuration of single atoms. Fig. 2 shows the change of the non-bonded radius and electronegativity of Fe with pressure relative to Si. These atoms are two of the most important constituents of the Earth's crust, mantle (*p* < 140 GPa), and core (*p* < 360 GPa), and are chosen here to illustrate just how radically different we can expect chemistry to be at different thermodynamic conditions. The sharp discontinuities predicted in both properties at 30 and 144 GPa coincide with transitions of the ground state electronic configuration of the Fe atom. Such transitions, exemplified for Fe in Fig. 2, are common to many alkali, alkaline earth, transition metal, and f-block atoms, and are well-known from both theory (*e.g.*, ref. 49, 62 and 63) and experiment (*e.g.*, ref. 53 and 64). Ground state configurational transitions in non-bonded atoms (and many materials) are isobaric processes, *i.e.*, at the transition pressure the atoms can have any of two well-defined sizes, energies, and electronegativities.

One criterion for transitioning between competing electronic states, such as those resulting in sharp discontinuities in Fig. 2, is for the difference in enthalpy between states at the transition pressure to vanish, *i.e.*, Δ*H* = 0. The electronic energy difference associated with such a pressure-induced electronic configurational transition is, however, non-zero. Since, in the absence of chemical reactions, the only source of energy is pressure–volume work, the difference in electronic energy at the transition pressure is equal to the negative of the *p*Δ*V*-term,^65^

4 Δ*E* = −*p*Δ*V*.

By combining eqn (3) and (4), we can express the electronic energy associated with compression of a non-reacting atom from a radius of *r*_1_ to *r*_2_ at pressure *p* as5
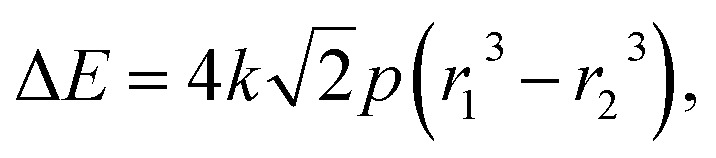
where Δ*E* is given in eV, *p* is the pressure in GPa, *r*_1_ and *r*_2_ are radii in Å, and *k* is a unit conversion factor equaling 6.242 × 10^−3^. By combining eqn (1) and (5) with the fact that for single-atom compression Δ*E*_NN_ = 0, the relationship between radius and electronegativity becomes6
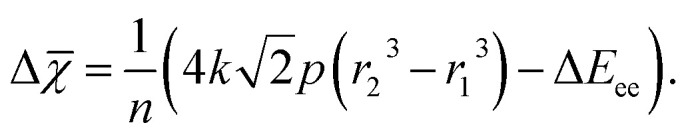


Eqn (6) tells us to expect decreases in electronegativity when the radius of an atom is decreased under constant pressure. Electronegativity also decreases as electron–electron repulsion, quantified by the Δ*E*_ee_-term, increases. We remind that eqn (6) has been derived for compression of non-reactive atoms. Arguments based on this equation are therefore not necessarily always applicable to other situations, such as volume changes quantified through, *e.g.*, experimental equations of state. Eqn (6) nevertheless helps us to understand how reduction of the oxidation state of an atom under ambient conditions (where the temperature is low and *p* ≈ 0) leads to a decrease in electronegativity (this happens as Δ*E*_ee_ > 0 for electron attachment). And *vice versa*, how oxidation of an atom (*i.e.*, where Δ*E*_ee_ < 0) leads to an increase in electronegativity. Care should be taken, however, not to use eqn (6) for explaining relative differences between atoms in the absence of a transition. In a static sense, highly electronegative atoms, such as F, will at any pressure be smaller than most other atoms, and consequently be subject to more electron–electron interactions.

### Interpreting changes in radii in terms of energy

We can use eqn (5) to attribute energies to volume contractions that are caused by electron configurational transitions in single atoms. Eqn (1) additionally reminds us that the decrease in electronegativity, or the average orbital destabilization, in such atoms upon compression does not equal the concomitant change in the total energy. The relationship between changes in energy and electronegativity includes a multiplication by the number of electrons *n*, and the subtraction of the non-trivially calculable electron–electron interactions described by the Δ*E*_ee_-term.

What electronic energy can be ascribed to the radial contractions shown in Fig. 2 (and the many others reported in ref. 4)? Eqn (5) shows that this depends on both the transition pressure and the radius of the atom. The larger the atom and the pressure, the larger is the energy attributed to a given radial contraction.

**Fig. 3:**
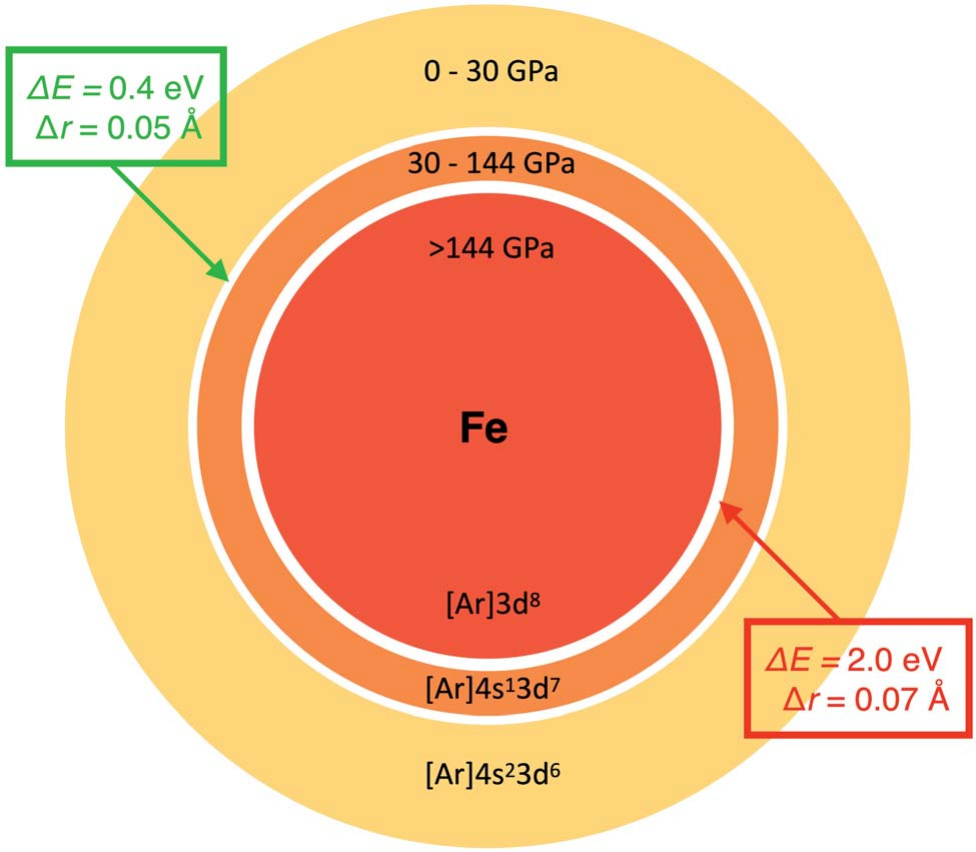
Example of the relationship between atomic radius and total energy. The size of the Fe atom at different pressures is to scale. White regions correspond to [Ar]4s^2^3d^6^ (*S* = 2) → [Ar]4s^1^3d^7^ (*S* = 2) and [Ar]4s^1^3d^7^ → [Ar]3d^8^ (*S* = 1) ground state configurational transitions predicted at 30 and 144 GPa, respectively.

In our example of Fe, the atom is predicted to contract its radius from 1.68 Å to 1.63 Å at a pressure of 30 GPa. In this case, the corresponding energy change, Δ*E*, *viz.*eqn (5), equals 0.43 eV (Fig. 3). In the second transition, the Fe atom shrinks by a similar magnitude from 1.38 Å to 1.31 Å. Because of a higher pressure at the second transition, the corresponding energy change is, however, considerably larger, 1.95 eV (Fig. 3).

It is important to note that the energies calculated for radial contractions should not be interpreted as the difference in energy between competing frontier orbitals, such as 4s *vs.* 3d in Fe.^50,51^ Small changes in radii also do not necessarily correspond to small concomitant changes in electronegativity or *vice versa*. In the case of Fe, the decrease in electronegativity, or average electron destabilization Δ*χ̄* in eqn (6), amounts to 4.7 and 5.5 eV e^−1^, for the two 4s → 3d transitions (Fig. 2).

**Fig. 4:**
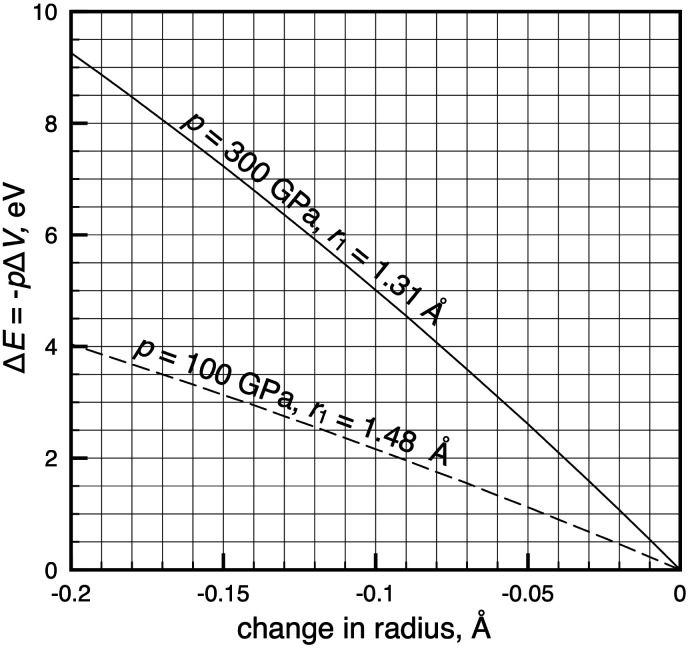
Energies associated with isobaric radial contractions in average-sized non-reacting atoms at two pressures. The dashed line shows radial contractions of the average atom at 100 GPa, for which the radius is 1.48 Å. The solid line shows radial contractions of the average atom at 300 GPa, for which the radius is 1.31 Å.

Fig. 4 allows for a quicker translation between isobaric radial contractions and energies at two high pressures, 100 and 300 GPa. Each curve in Fig. 4 describes compression of an atom of average size, which in our data set of 93 atoms corresponds to a radius of 1.48 Å and 1.31 Å at *p* = 100 and 300 GPa, respectively. Fig. 4 tells us that radial contractions calculated for atoms in the investigated pressure range^4^ correspond to energies that are large enough to be chemically relevant, but that are smaller than 3.5 eV.

Above we have focused our discussion on the electronic transitions in Fe, but we will give a few other examples for comparison: in our model, K is predicted to undergo a 4s → 3d transition at 56 GPa and, as a consequence, contract its radius from 1.62 Å to 1.47 Å.^4^ The corresponding energy change, Δ*E*, equals 2.06 eV, while Δ*χ̄* equals 3.8 eV e^−1^.

The largest effect of an s → d transition in the d-block is predicted for Cr, where a contraction by 0.08 Å at 208 GPa translates into an electronic energy increase of 3.0 eV with Δ*χ̄* = 5.8 eV e^−1^. Another example is Ce, which is predicted to undergo a small radial contraction by 0.02 Å at 270 GPa. The Ce contraction translates to a 1.0 eV change in total energy. However, the predicted corresponding decrease in electronegativity of Ce at this transition pressure is much larger at 5.5 eV e^−1^. The disconnect between atomic size and electronegativity is noticeably larger for the f-block atoms, a fact we attribute to more substantial changes in their electron–electron interactions (the Δ*E*_ee_-term in eqn (6)) during isobaric transitions, compared to the lighter elements. In other words, chemical intuition, which is often aided by atomic properties such as radius and electronegativity, is even harder to come by for the heaviest atoms.

## Conclusions

The picture that emerges from our analysis is arguably one in which atomic radius and electronegativity walk hand-in-hand; both properties decrease with pressure relative to ambient conditions. However, relative differences in these atomic properties may both increase or decrease with a perturbation such as compression.^4,49^

Following the calculation of radius and electronegativity as a function of pressure in past work,^4,49^ we here derive eqn (6) that connects the two quantities in isobaric transformations of non-bonded atoms. The framework we have outlined can pave the way for a more general understanding of these central chemical concept with wider implications in chemistry and materials science. In the end, these atomic properties, no matter how defined or quantified, are merely approximations and guides to the behavior of real bonded materials. Detailed analyses and consideration of electronic structure will always be necessary for quantitative evaluations of, *e.g.*, bond strength and polarity.^66,67^

## Author contributions

Conceptualization (MR), writing – original draft (MR, PE, RC), data curation (MR, PE), software (RC, PE, MR).

## Conflicts of interest

There are no conflicts to declare.
